# CircCDC45 promotes the malignant progression of glioblastoma by modulating the miR-485-5p/CSF-1 axis

**DOI:** 10.1186/s12885-021-08803-7

**Published:** 2021-10-09

**Authors:** Rongcai Liu, Weimin Dai, An Wu, Yunping Li

**Affiliations:** Department of Neurosurgery, Quzhou People’s Hospital, No. 2, Zhongloudi, Kecheng District, Quzhou, 324000 Zhejiang China

**Keywords:** circCDC45, miR-485-5p, CSF-1, Glioblastoma

## Abstract

**Background:**

Glioblastoma (GBM) is characterized by progressive growth and metastasis. Numerous studies claim that the deregulation of circular RNAs (circRNAs) is associated with cancer progression. However, the role of circRNAs in GBM is largely limited. The purpose of this study was to investigate the functions of circCDC45 in GBM and provide a feasible functional mechanism to support its role.

**Methods:**

The expression of circCDC45, miR-485-5p and colony-stimulating factor 1 (CSF-1) mRNA was examined using quantitative real-time polymerase chain reaction (qRT-PCR). Cell proliferation was assessed using cell counting kit − 8 (CCK-8) assay and colony formation assay. Cell migration and cell invasion were monitored using transwell assay. The protein levels of proliferation-related markers and CSF-1 were determined using western blot. The target relationship was predicted using bioinformatics tools and validated using dual-luciferase reporter assay and RNA immunoprecipitation (RIP) assay. Animal models were constructed to verify the role of circCDC45 in vivo.

**Results:**

The expression of circCDC45 and CSF-1 was elevated in GBM tissues and cells, while the expression of miR-485-5p was declined. Downregulation of circCDC45 or CSF-1 blocked GBM cell proliferation, invasion and migration as well as tumor growth in vivo. In mechanism, circCDC45 positively regulated the expression of CSF-1 by targeting miR-485-5p. Inhibition of miR-485-5p reversed the biological effects caused by circCDC45 downregulation in GBM cells.

**Conclusion:**

CircCDC45 promoted the progression of GBM by mediating the miR-485-5p/CSF-1 axis, and circCDC45 might be a promising plasmatic biomarker for GBM diagnosis and treatment.

**Supplementary Information:**

The online version contains supplementary material available at 10.1186/s12885-021-08803-7.

## Background

Glioblastoma (GBM) is one of the common primary brain tumors [1]. The median survival time of GBM patients is only about 14.6 months, and almost all patients have a poor prognosis [2]. GBM, grade IV astrocytoma, is derived from multiple cell types with neural stem cell-like properties, and it is almost unfulfillable to completely remove the entire GBM by surgery [3, 4]. Partial GBM cells infiltrating into nearby brain tissues can easily cause tumor recurrence [3, 5]. So GBM is considered to be one of the most aggressive and most difficult cancers to treat [6]. The identification and verification of prognosis and predictive biomarkers are of great significance for the understanding of tumor molecular characteristics and targeted therapy.

Circular RNAs (circRNAs) are characterized by a covalently closed loop with no free 3′ or 5′ end and belong to non-coding RNAs [7]. Emerging research reports that numerous circRNAs are deregulated in human cancers and closely modulate the progression of cancers [8]. CircRNAs can functionally either act as oncogenes or tumor suppressors in cancers with abundant and stable expression [7, 8] and thus are defined as effective biomarkers for cancer diagnosis and therapy [9]. CircRNA microarray identified a series of differently expressed circRNAs in GBM tissues compared to normal tissues [10, 11], which provided candidates for the following analyses. Although the vital role of circRNAs has been recognized, research on the function of circRNAs in cancer is still limited. Here, circCDC45, which was identified to be highly expressed in GBM tissues [11], caught our attention. CircCDC45, also named circ_0062270, was derived from CDC45 mRNA. The role and functional mechanism of circCDC45 in GBM are unknown and need to be further explored.

Competitive endogenous RNAs (ceRNAs) compete for microRNA (miRNA) response elements (MREs) to mediate the expression of miRNAs and downstream miRNA target genes [12]. Numerous studies demonstrated that circRNAs could serve as ceRNAs to mediate gene expression, which developed to be a canonical mechanism of circRNAs function in human cancers [13, 14]. MiR-485-5p was reported to play an anti-tumor role in glioma to block glioma cell proliferation and invasion [15]. Previous studies also mentioned that circAMOTL1 competed for miR-485-5p, regulating the development of cervical cancer [16]. However, the interaction between circCDC45 and miR-485-5p is not presented and is worth exploring.

MiRNAs generally combine with the 3′ untranslated regions (3’UTR) of target mRNAs, triggering mRNA degradation and translation inhibition [17]. The 3’UTR of colony-stimulating factor 1 (CSF-1) harbors multiple unique motifs, and CSF-1 acting as a target of various miRNAs has been documented in cancers [18, 19]. In addition, CSF-1 was extensively reported in glioma, involving in ionizing radiation resistance, high-grade of glioma, cell proliferation and invasion [20, 21]. Novel action mechanism of CSF-1 linked to miR-485-5p helps to enrich the role of CSF-1 in GBM.

Here, we investigated the function of circCDC45 in GBM both in vitro and in vivo. Besides, we predicted and validated the interaction between miR-485-5p and circCDC45 or CSF-1, thus providing a circCDC45/miR-485-5p/CSF-1 regulatory network to illustrate the function mechanism of circCDC45 in GBM. We aimed to provide promising biomarkers and strategies for GBM diagnosis and treatment.

## Methods

### Specimen collection

GBM specimens (*n* = 40) were collected from GBM patients, and normal brain tissues (NBTs) (*n* = 40) were collected from patients who underwent brain tissue resection of craniocerebral injury. All subjects were recruited from Quzhou People’s Hospital. Besides, equal amount of plasma specimens was also collected from each subject, and plasma specimens from GBM patients before and after surgical resection (Pre-operation and Post-operation) were collected separately. Informed consent for specimen use was obtained from each participant. All specimens were promptly treated using liquid nitrogen after excising and then preserved at − 80 °C conditions. This study was carried out with the approval of the Ethics Committee of Quzhou People’s Hospital.

### Cell lines

GBM cell lines, including U251, LN229, SHG-44, A172 and T98, and normal human astrocytes (NHA) were purchased from Bena Culture Collection (Beijing, China) and Procell Co., Ltd. (Wuhan, China). According to the culture method, all cells were cultured in 90% Dulbecco’s Modified Eagle Medium (DMEM; Gibco, Grand Island, NY, USA) plus 10% fetal bovine serum (FBS; Gibco) in an incubator at 37 °C containing 5% CO_2_.

### Quantitative real-time polymerase chain reaction (qRT-PCR)

Total RNA from specimens and cells was extracted using the Total RNA Kit (TianGen, Beijing, China). The synthesis of cDNA was performed using the cDNA first-strand synthesis kit (TianGen). For miRNA cDNA synthesis, reverse transcription was carried out using the miRcute miRNA cDNA first-strand synthesis kit (TianGen). Subsequently, qRT-PCR was implemented using the Talent qPCR SYBR Green Mix (TianGen). All experimental procedures were conducted following the protocols. The conditions used for qRT-PCR were as it follows: 95 °C for 10 min (1 cycle), 95 °C for 10 s and 60 °C for 1 min (40 cycles). Relative expression was computed using the 2^-ΔΔCT^ means with GAPDH or U6 as a house-keeping gene. The primer sequences were listed as follows: circCDC45, F: 5′-TTTGCACCAACCTCGTCATC-3′ and R: 5′-GTCCTTCATCCGAACACACA-3′; CDC45, F: 5′-GAGTGGCTCTGGGAGTGAAC-3′ and R: 5′-GGCTGACGATGTCCCATGAT-3′; miR-485-5p, F: 5′-GGAGAGGCTGGCCGTGAT-3′ and R: 5′-CAGTGCGTGTCGTGGAGT-3′; CSF-1, F: 5′-ACCCCTCCACCCTCTCTG-3′ and R: 5′-CTGCCCCTTCACTTGCTG-3′; GAPDH, F: 5′-AAGTATGACAACAGCCTCAAGA-3′ and R: 5′-CACCACCTTCTTGATGTCATCA-3′; U6, F: 5′-CTCGCTTCGGCAGCACA-3′ and R: 5′-AACGCTTCACGAATTTGCGT-3′.

### Actinomycin D treatment

GBM cells were treated with Actinomycin D (50 ng/mL; Cell Signaling Technology, Danvers, MA, USA) and maintained for 8, 16 and 24 h at room temperature. Cells at different time points were harvested and used for qRT-PCR to monitor the expression of circCDC45 and CDC45 mRNA. Three independent experiments were conducted.

### RNase R digestion

The isolated RNAs from GBM cells were exposed to RNase R (2 U/μg RNA, Epicentre, Madison, WI, USA) for 15 min at 37 °C. Then, total RNA was used for qRT-PCR analysis to detect the expression of circCDC45 and CDC45 mRNA. Untreated RNA (Mock) was used as control. Three independent experiments were conducted.

### Cell transfection

Small interference RNA (siRNA) was used for expression silencing. In brief, siRNA targeting circCDC45 (si-circCDC45#1 and si-circCDC45#2), siRNA targeting CSF-1 (si-CSF-1#1 and si-CSF-1#2) and siRNA negative control (si-NC) were constructed by GenePharma (Shanghai, China). pCDNA3.1(+) CircRNA Mini Vector was used for circCDC45 overexpression (pcDNA-circCDC45), and fusion plasmid was constructed by Ke Lei BioTech Co., Ltd. (Shanghai, China) with empty pcDNA vector as a negative control (pcDNA-NC). MiR-485-5p mimics (miR-485-5p), miR-485-5p inhibitors (anti-miR-485-5p) and their separate control (miR-NC and anti-miR-NC) were all obtained from Ribobio (Guangzhou, China). The oligonucleotides (siRNA: 200 nM; mimic and inhibitor: 100 nM) or fusion plasmids (1 μg) were transfected into cells using Lipofectamine 3000 (Invitrogen, Carlsbad, CA, USA) in line with detailed usage instructions.

### Cell counting kit-8 (CCK-8) assay

GBM cells were plated in 96-well plates (2000 cells/well) with different transfection. After culturing for 24, 48 and 72 h, cells were exposed to 10 μL CCK-8 reagent (Beyotime, Shanghai, China) and cultured for continuing 4 h. The absorbance at 450 nm of GBM cells at different time points was examined using a microplate reader (Bio-Rad, Hercules, CA, USA). Each sample was in triplicate, and three independent experiments were conducted.

### Colony formation assay

GBM cells were cultured in 6-well plates (10 cm diameter) with different transfection, at a density of 200 cells/plate. The plates were cultured at 37 °C conditions containing 5% CO_2_. After 2 weeks, formed colonies were fixed using methanol and then stained using 0.1% crystal violet followed by photograph under a microscope (Olympus, Tokyo, Japan). Each sample was in triplicate, and three independent experiments were conducted.

### Western blot

Total proteins were extracted using RIPA Lysis Buffer (Beyotime) and next separated by 10% sodium dodecyl sulfate-polyacrylamide gel electrophoresis (SDS-PAGE). The separated proteins were transferred onto polyvinylidene difluoride (PVDF) membranes (Bio-Rad) and incubated with the blocking buffer. Subsequently, the membranes containing protein bands were probed with the primary antibodies against Ki67 (ab231172; Abcam, Cambridge, MA, USA), Proliferating cell nuclear antigen (PCNA; ab18197; Abcam), CSF-1 (ab99178; Abcam) and GAPDH (ab9485; Abcam) at 4 °C overnight and subsequently treated with the secondary antibodies (ab205718; Abcam). The protein bands were imaged using an enhanced chemiluminescence kit (ECL; Beyotime). Each sample was in triplicate, and three independent experiments were conducted.

### Transwell assay

Cell migration and cell invasion capacities were monitored using transwell chambers (BD Biosciences, San Jose, CA, USA). For invasion analysis, GBM cells (5 × 10^4^ cells) in 150 μL serum-free medium were planted into the top of chambers pre-coated with Matrigel (BD Biosciences). For migration analysis, GBM cells (1 × 10^4^ cells) in serum-free medium were added into the upper chambers with nothing. Meantime, 600 μL fresh DMEM containing 10% FBS was filled with the lower chambers. After 24-h incubation, cells remaining on the upper membrane were removed with a cotton swab, and the lower cells were fixed in methanol and then stained using 0.1% crystal violet followed by observation and photograph using a microscope (Olympus) with 5 randomly selected fields (magnification 100×). Each sample was in triplicate, and three independent experiments were conducted.

### Bioinformatics analysis

We used the bioinformatics databases, including starBase (http://starbase.sysu.edu.cn/) and circbank (http://www.circbank.cn/), to predict the potential miRNAs targeted by circCDC45, and starBase was utilized to predict the potential mRNAs targeted by miR-485-5p.

### Dual-luciferase reporter assay

Luciferase reporter plasmids were assembled in pmirGLO vector (Promega, Madison, WI, USA) containing the wild-type sequence of circCDC45 (harboring miR-485-5p binding sites) or containing the mutant-type sequence of circCDC45 (harboring mutated miR-485-5p binding sites). The fusion plasmids were named as circCDC45-WT and circCDC45-MUT, respectively. Similarly, the fusion plasmids, CSF-1 3’UTR-WT and CSF-1 3’UTR-MUT were also generated in the same method. LN229 and A172 cells were then cotransfected with miR-485-5p (50 nM) or miR-NC (50 nM) together with circCDC45-WT, circCDC45-MUT, CSF-1 3’UTR-WT and CSF-1 3’UTR-MUT (200 μg) using Lipofectamine 3000 and cultured for 48 h. Luciferase activity was ascertained in LN229 and A172 cells using the Dual-Luciferase Assay System (Promega). Three independent experiments were conducted.

### RNA immunoprecipitation (RIP) assay

LN229 and A172 cells were lysed using the lysis buffer from the Magna RIP Kit (Millipore), and cell lysates were then incubated with RIP buffer containing magnetic beads conjugated with Argonaute 2 antibody (anti-Ago2) or Immunoglobulin G antibody (anti-IgG; control) at 4 °C overnight. Afterwards, RNA was eluted and purified, and immunoprecipitated RNA was analyzed by qRT-PCR. Three independent experiments were conducted.

### In vivo experiments

A total of 12 BALB/C nude mice (Male, 4–6-week-old, 16–20 g) were purchased from Beijing HFK Bioscience Co., Ltd. (Beijing, China) and randomly divided into 2 groups (*n* = 6 per group). Short hairpin RNA (shRNA) lentiviral vector containing circCDC45 (sh-circCDC45), and its negative controls (sh-NC) were synthesized by GeneCopoeia (Guangzhou, China). LN229 cells (100 μL; 1 × 10^6^ cells) transfected with sh-circCDC45 or sh-NC were subcutaneously implanted into nude mice. Mice were adaptively raised for 1 week to allow tumor growth. Then, tumor volume was recorded once a week, lasting 6 weeks. After 6 weeks, all mice were sacrificed to collect tumor tissues for the following analysis. All animal experimental procedures were approved by the Animal Care and Use Committee of Quzhou People’s Hospital.

### Statistical analysis

All experiments were conducted at least three times. Differences between two groups were analyzed by Student’s *t*-test, and differences among ≥ three groups were analyzed by analyses of variance with Tukey post hoc test. The expression correlation in clinical tissues between two variables was performed using Spearman’s correlation test. Statistical analyses were carried out using GraphPad Prism 7 (GraphPad Software, Inc., La Jolla, CA, USA). *P* value ≤0.05 was deemed to be statistically significant. The data were shown as the mean ± standard deviation.

## Result

### CircCDC45 was aberrantly highly expressed in GBM tissues and cells

**Fig. 1:**
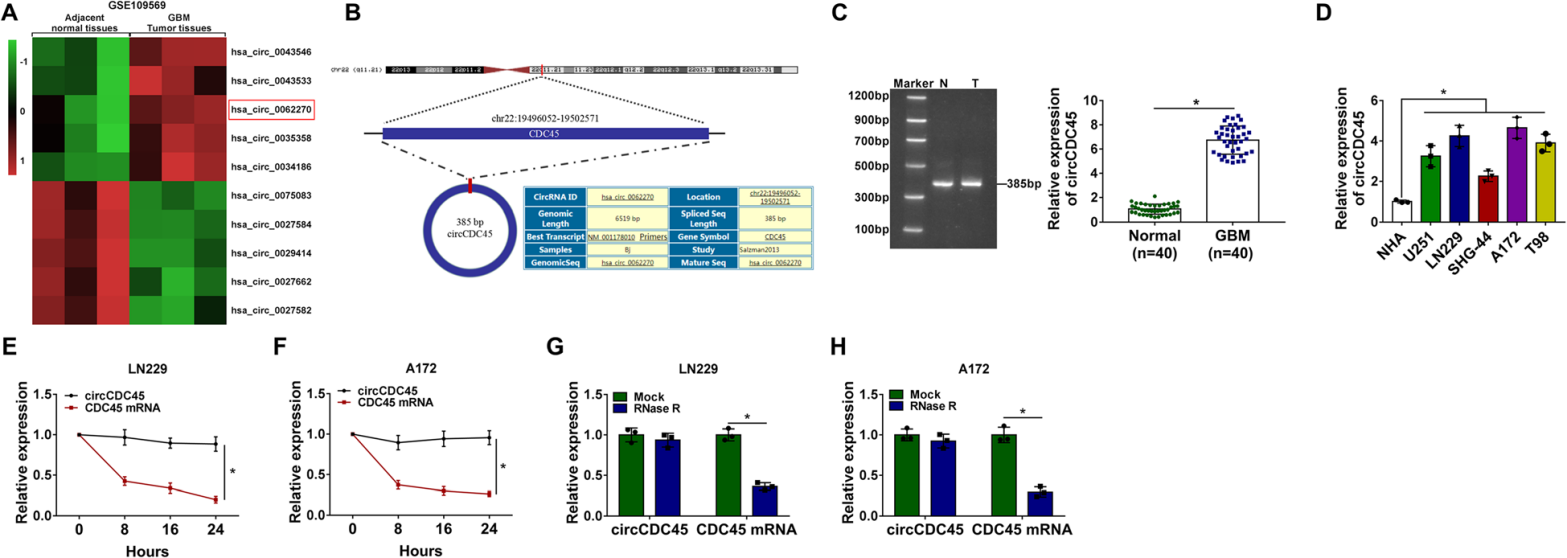
The expression of circCDC45 was elevated in GBM tissues and cells**.** (**A**) Heap map based on the GEO dataset (GSE109569) revealed that circCDC45 was differently expressed in GBM tissues and normal tissues. (**B**) The schematic diagram was depicted to describe the formation of circCDC45. (**C**) The identification and expression of circCDC45 in clinical tissues was determined by PCR and qRT-PCR. (**D**) The expression of circCDC45 in GBM cell lines and normal cell lines was detected by qRT-PCR. (**E** and **F**) The stability of circCDC45 was verified by Actinomycin D. (**G** and **H**) The stability of circCDC45 was verified by RNase R. *P < 0.05

The microarray data of circRNAs that were differently expressed in GBM tissues and non-tumor tissues were deposited in the Gene Expression Omnibus (GEO) database (accession: GSE109569; https://www.ncbi.nlm.nih.gov/geo/query/acc.cgi?acc=GSE109569), and we downloaded the data and analyzed the top 5 circRNAs significantly downregulated or upregulated in GBM tissues. Heat map showed that circCDC45 (has_circ_0062270) is one of the significantly upregulated circRNAs in GBM tissues (Fig. 1A). CircCDC45 was derived from linear CDC45 mRNA, and the schematic diagram was depicted to describe the formation of circCDC45 (Fig. 1B). The size of circCDC45 in tumor tissues and normal tissues was determined by PCR, and a band with about 385 bp was presented in gel (Fig. 1C). Then we verified the expression of circCDC45 in clinical specimens by qRT-PCR, and the data displayed that the expression of circCDC45 was noticeably promoted in GBM tissues (*n* = 40) compared with that in NBT tissues (*n* = 40) (Fig. 1C). Likewise, the expression of circCDC45 was also elevated in GBM cell lines (U251, LN229, SHG-44 and T98) compared with that in normal glial cells (NHA) (Fig. 1D). LN229 and A172 cells were chosen for the following in vitro analyses because the expression of circCDC45 in these two cell lines was relatively higher than other GBM cell lines. Besides, Actinomycin D treatment notably weakened the expression of CDC45 mRNA expression but had negligible effects on circCDC45 expression (Fig. 1E and F). RNase R treatment also notably impaired the expression of CDC45 mRNA expression but hardly weakened the expression of circCDC45 (Fig. 1G and H). These data hinted that circCDC45 was dysregulated in GBM and might take part in GBM progression.

### CircCDC45 downregulation blocked GBM cell proliferation, migration and invasion

**Fig. 2:**
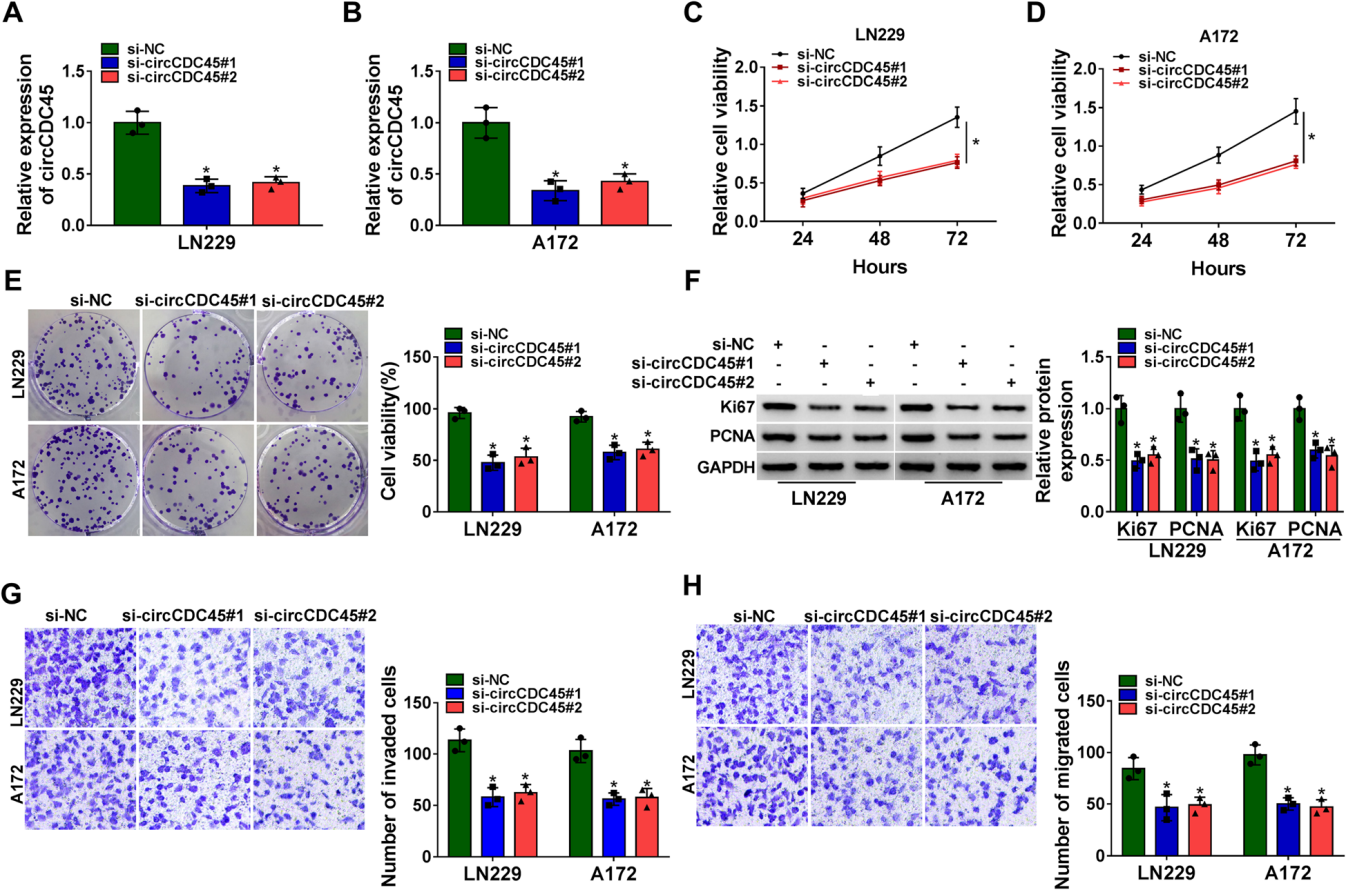
CircCDC45 downregulation suppressed proliferation, migration and invasion of GBM cells. siRNA targeting circCDC45 was used for circCDC45 downregulation in LN229 and A172 cells. (**A** and **B**) The expression of circCDC45 was measured by qRT-PCR. (**C** and **D**) Cell proliferation was assessed by CCK-8 assay. (**E**) Cell proliferation was also assessed by colony formation assay. (**F**) The expression of Ki67 and PCNA was quantified by western blot. (**G** and **H**) Cell invasion and migration were investigated by transwell assay. *P < 0.05

Loss-function of circCDC45 was conducted to explore the role of circCDC45 in GBM cells. After the transfection of si-circCDC45#1 or si-circCDC45#2, the expression of circCDC45 was significantly declined in LN229 and A172 cells (Fig. 2A and B). CircCDC45 downregulation markedly repressed GBM cell proliferation by the analyses of CCK-8 and colony formation (Fig. 2C, D and E). Besides, cell proliferation-related proteins, including Ki67 and PCNA, were quantified, and the data showed that both Ki67 and PCNA were significantly downregulated in LN229 and A172 cells transfected with si-circCDC45 (Fig. 2F). The raw images of western blot data for the bands of Ki67 and PCNA were shown in Additional file 1: Supplementary file. CircCDC45 downregulation also suppressed GBM cell invasion and migration by transwell assay (Fig. 2G and H). These data suggested that circCDC45 downregulation inhibited GBM malignant behaviors in vitro.

### CircCDC45 functioned as the molecular sponge of miR-485-5p

**Fig. 3:**
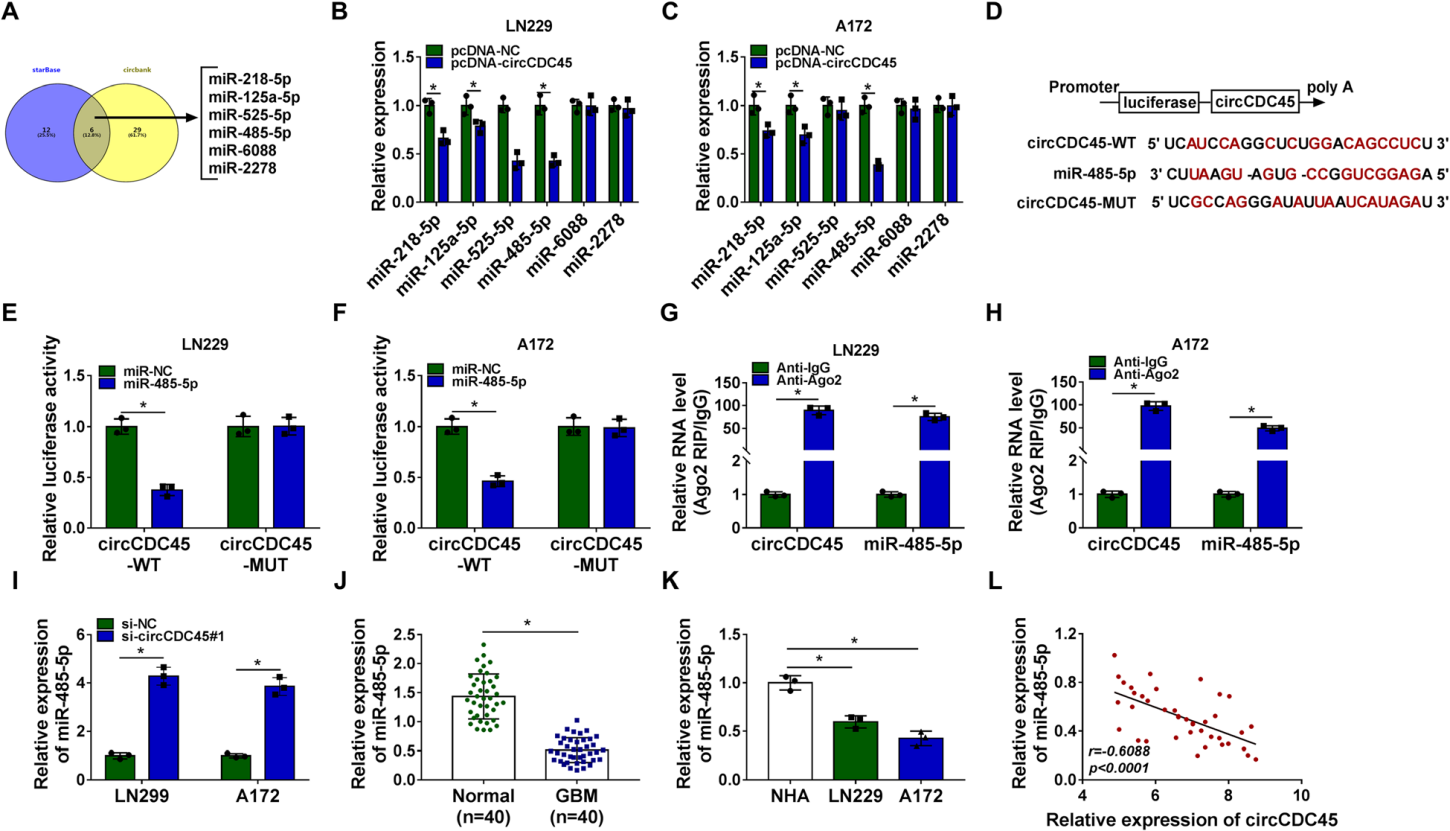
MiR-485-5p was one of the targets of circCDC45. (**A**) The miRNAs targeted by circCDC45 was predicted by the online tools starBase and circbank. (**B** and **C**) The expression of predicted miRNAs in LN229 and A172 cells with circCDC45 overexpression was detected by qRT-PCR. (**D**) The wild and mutant circCDC45 sequence fragments were amplified and cloned into luciferase reporter plasmid. (**E** and **F**) The relationship between circCDC45 and miR-485-5p was validated by dual-luciferase reporter assay. (**G** and **H**) The relationship between circCDC45 and miR-485-5p was validated by RIP assay. (**I**) The expression of miR-485-5p in LN229 and A172 cells with circCDC45 knockdown was detected by qRT-PCR. (**J** and **K**) The expression of miR-485-5p in clinical tissues and cell lines was also detected by qRT-PCR. (**L**) Spearman’s correlation analysis revealed the correlation between miR-485-5p expression and circCDC45 expression in GBM tissues. *P < 0.05

The view that certain circRNAs can function as decoys or sponges to suppress miRNA expression and degrade miRNA function is canonical [22]. To identify that circCDC45 harbored the same property, we predicted the potential target miRNAs using starBase and circbank. A total of 18 miRNAs targeted by circCDC45 were obtained from starBase, and a total of 35 miRNAs targeted by circCDC45 were obtained from circbank, including 6 miRNAs (miR-218-5p, miR-125a-5p, miR-525-5p, miR-485-5p, miR-6088 and miR-2278) in both starBase and circbank (Fig. 3A). We focused on these 6 miRNAs and examined their expression in LN229 and A172 cells with circCDC45 overexpression. Interestingly, the expression of miR-485-5p decreased most (Fig. 3B and C). We deduced that miR-485-5p was likely a target of circCDC45. To further validate the interaction between miR-485-5p and circCDC45, the mutant sequence of circCDC45 with miR-485-5p binding sites was generated according to its wild sequence (Fig. 3D). Dual-luciferase reporter assay showed that a decrease of luciferase activity was observed in LN229 and A172 cells with cotransfection of miR-485-5p and circCDC45-WT (Fig. 3E and F). In addition, RIP assay presented that both circCDC45 and miR-485-5p were significantly enriched in the Anti-Ago2 group compared with that in the Anti-IgG group (Fig. 3G and H). In LN229 and A172 cells with circCDC45 knockdown, the expression of miR-485-5p was remarkably enhanced (Fig. 3I). Moreover, the expression of miR-485-5p was remarkably declined in GBM clinical specimens and GBM cell lines compared with that in NBT specimens and NHA cells, respectively (Fig. 3J and K), and miR-485-5p expression was negatively correlated with circCDC45 expression in GBM clinical tissues (Fig. 3L). All data manifested that miR-485-5p was a target of circCDC45, and the expression pattern of miR-485-5p in GBM was opposite to circCDC45.

### CircCDC45 regulated GBM cell proliferation, migration and invasion by targeting miR-485-5p

**Fig. 4:**
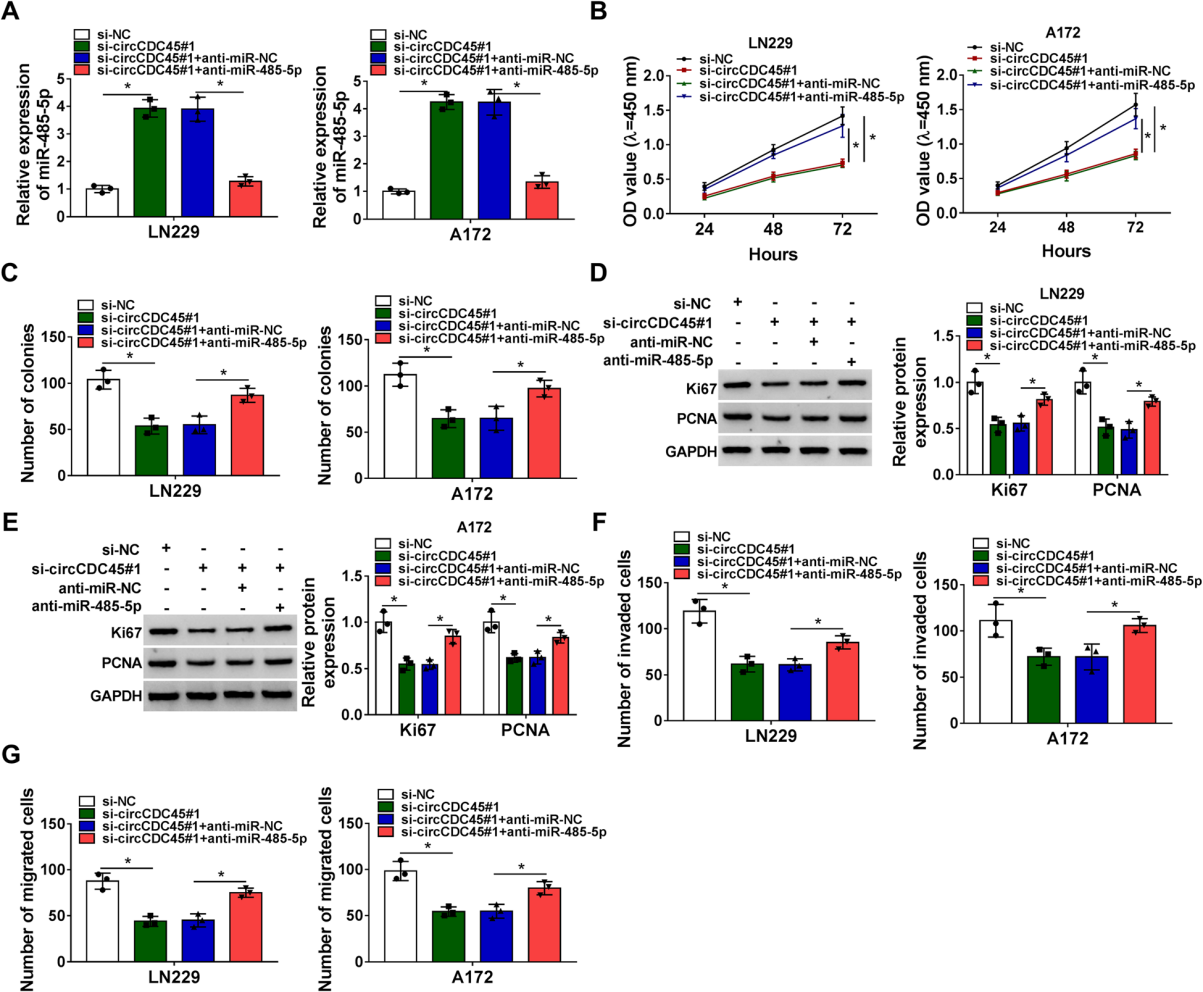
MiR-485-5p inhibition reversed the effects of circCDC45 downregulation**.** LN229 and A172 cells were transfected with si-circCDC45 or si-circCDC45 + anti-miR-485-5p with si-NC or si-circCDC45 + anti-miR-NC as the separate control. (**A**) The expression of miR-485-5p in these transfected cells was examined using qRT-PCR. (**B** and **C**) Cell proliferation was assessed by CCK-8 assay and colony formation assay. (**D** and **E**) The expression of Ki67 and PCNA was quantified by western blot. (**F** and **G**) Cell invasion and cell migration were investigated by transwell assay. *P < 0.05

LN229 and A172 cells with circCDC45 knockdown alone or circCDC45 and miR-485-5p knockdown together were used to test the contrary role of circCDC45 and miR-485-5p. The expression of miR-485-5p was significantly promoted in LN229 and A172 cells transfected with si-circCDC45 but significantly lessened in cells transfected with si-circCDC45 + anti-miR-485-5p (Fig. 4A). In function, OD value and colony number were blocked by circCDC45 downregulation, while the inhibition of miR-485-5p substantially recovered OD value and colony number (Fig. 4B and C). The expression of Ki67 and PCNA impaired in LN229 and A172 cells transfected with si-circCDC45 was largely restored in cells transfected with si-circCDC45 + anti-miR-485-5p (Fig. 4D and E). The number of invaded and migrated cells was weakened by circCDC45 downregulation alone but promoted by circCDC45 downregulation combined with miR-485-5p inhibition in LN229 and A172 cells (Fig. 4F and G). The data suggested that miR-485-5p inhibition could promote GBM cell malignant behaviors blocked by circCDC45 downregulation.

### CSF-1 was a target of miR-485-5p

**Fig. 5:**
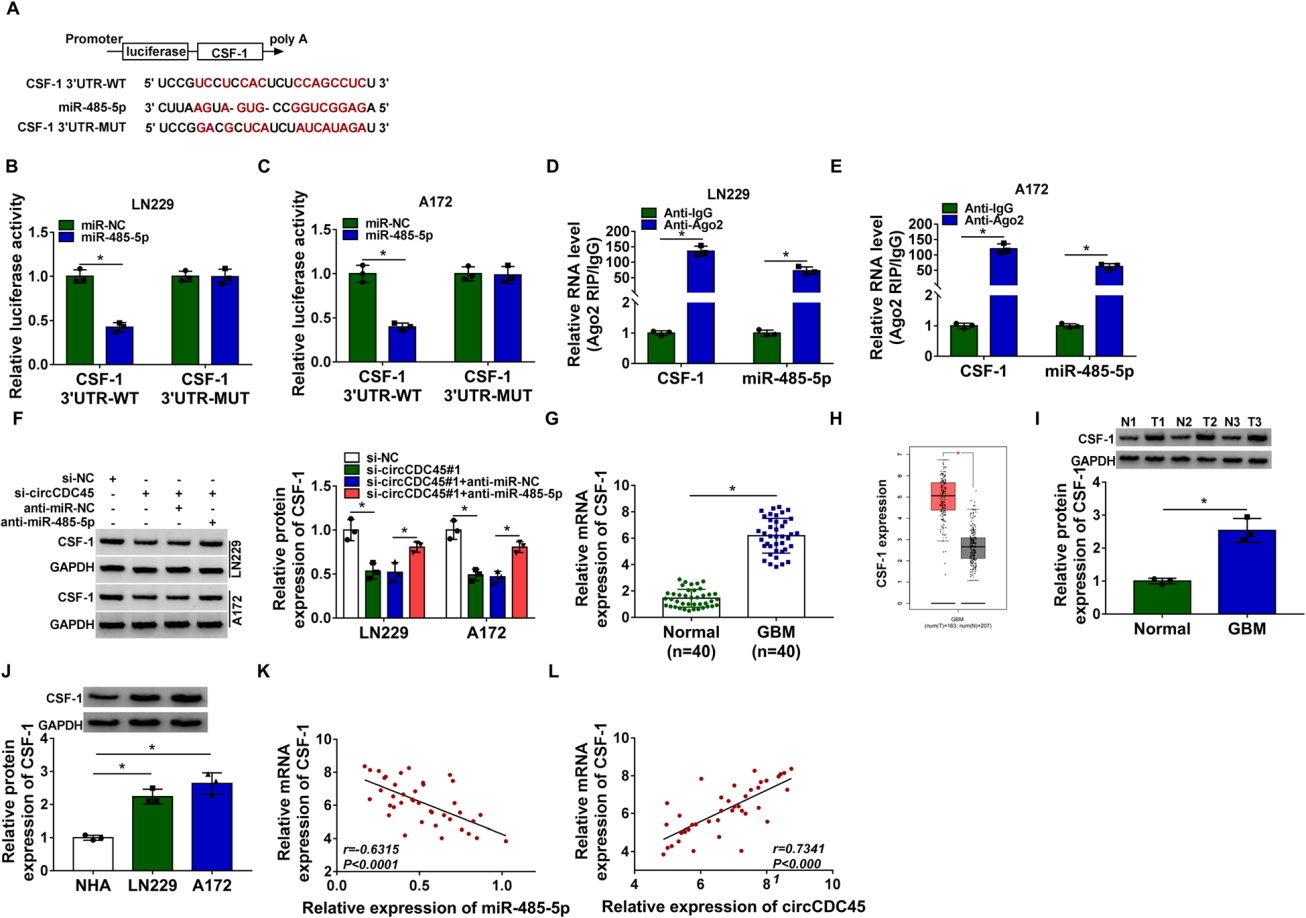
MiR-485-5p directly bound to CSF-1. (**A**) CSF-1 was predicted as a target of miR-485-5p by starBase, and the wild and mutant sequences of CSF-1 3’UTR were designed for dual-luciferase reporter assay. (**B** and **C**) The interaction between LN229 and A172 was validated by dual-luciferase reporter assay. (**D** and **E**) The interaction between LN229 and A172 was validated by RIP assay. (**F**) The expression of CSF-1 in LN229 and A172 cells transfected with si-circCDC45, si-NC, si-circCDC45 + anti-miR-485-5p or si-circCDC45 + anti-miR-NC was checked by western blot. (**G**) The expression of circCDC45 in clinical tissues was checked using qRT-PCR. (**H**) The expression of circCDC45 in GBM tissues was obtained from the TCGA database. (**I**) The expression of circCDC45 in clinical specimens was checked by western blot. (**J**) The expression of circCDC45 in NHA, LN229 and A172 cells was examined using western blot. (**K** and **L**) Spearman’s correlation analysis revealed the correlation between CSF-1 expression and miR-485-5p expression or circCDC45 expression in GBM tissues. *P < 0.05

MiRNAs generally bound to the 3′ untranslated region (3’UTR) of mRNAs to post-transcriptionally regulated miRNA expression. Therefore, we predicted the potential mRNAs targeted by miR-485-5p, and the tool starBase exhibited that there were binding sites between miR-485-5p and CSF-1 3’UTR (Fig. 5A). The mutant sequence of CSF-1 3’UTR and the wild sequence of CSF-1 3’UTR were amplified and cloned into luciferase reporter plasmid, and we found miR-485-5p restoration strikingly reduced the luciferase activity of LN229 and A172 cells transfected with CSF-1 3’UTR-WT (Fig. 5B and C). RIP assay presented that both miR-485-5p and CSF-1 were enriched in the Anti-Ago2 group compared with that in the Anti-IgG group (Fig. 5D and E). In addition, we found that the expression of CSF-1 was decreased in LN229 and A172 cells transfected with si-circCDC45 but promoted in cells transfected with si-circCDC45 + anti-miR-485-5p (Fig. 5F), suggesting that circCDC45 could regulate the expression of CSF-1 by sponging miR-485-5p. Moreover, the expression of CSF-1 was significantly enhanced in GBM tissues (*n* = 40) compared with that in the NBTs (n = 40), which was consistent with the data from TCGA database (Fig. 5G, H and I). Likewise, the expression of CSF-1 was elevated in LN229 and A172 cells compared with that in NHA cells (Fig. 5J). Correlation analysis revealed that CSF-1 expression was negatively correlated with miR-485-5p expression but positively correlated with circCDC45 expression in GBM tissues (Fig. 5K and L). The data suggested that CSF-1 was a target of miR-485-5p, and circCDC45 regulated CSF-1 by targeting miR-485-5p.

### CSF-1 knockdown blocked GBM cell proliferation, migration and invasion

**Fig. 6:**
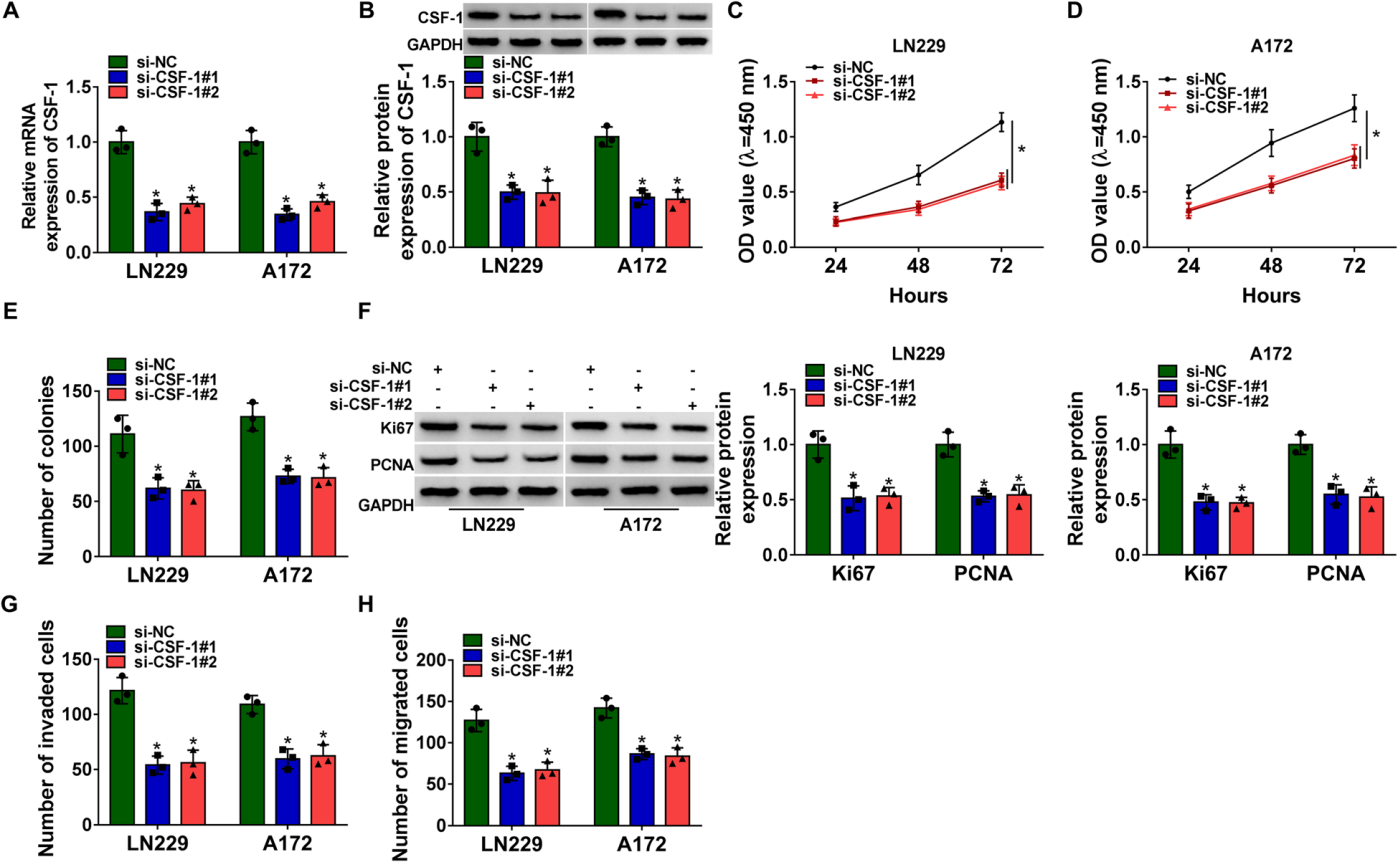
CSF-1 knockdown blocked GBM cell proliferation, migration and invasion. LN229 and A172 cells were transfected with si-CSF-1#1, #2 or si-NC. (**A** and **B**) The expression of CSF-1 in transfected cells was detected using qRT-PCR and western blot. (**C**, **D** and **E**) Cell proliferation was assessed by CCK-8 assay and colony formation assay. (**F**) The expression of Ki67 and PCNA was checked using western blot. (**G** and **H**) Cell invasion and cell migration were determined by transwell assay. *P < 0.05

The expression of CSF-1 was notably decreased in LN229 and A172 cells transfected with si-CSF-1#1 or si-CSF-1#2 (Fig. 6A and B). Then, LN229 and A172 cells with CSF-1 knockdown were used for functional analysis. CCK-8 assay and colony formation assay indicated that CSF-1 knockdown strikingly restrained cell proliferation (Fig. 6C, D and E). The expression of Ki-67 and PCNA was notably decreased in cells with CSF-1 knockdown (Fig. 6F). Moreover, transwell assay displayed that the ability of cell migration and invasion were restricted by CSF-1 knockdown (Fig. 6G and H). All data suggested that CSF-1 might be an oncogene in GBM.

### CircCDC45 downregulation repressed tumor growth in vivo by targeting the miR-485-5p/CSF-1 axis

**Fig. 7:**
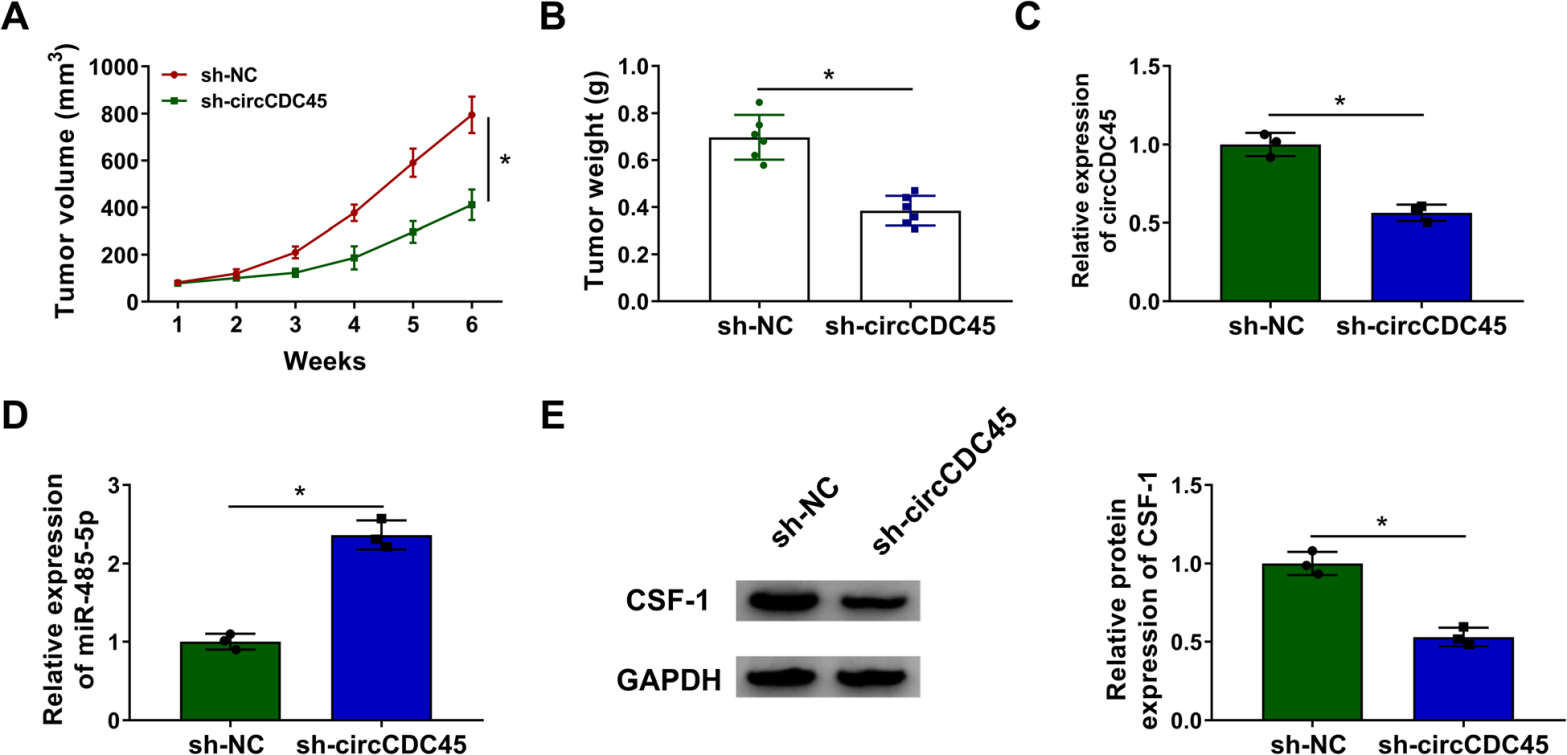
CircCDC45 regulated tumor growth in vivo by targeting the miR-485-5p/CSF-1 axis. (**A** and **B**) The effects of circCDC45 knockdown on tumor volume and tumor weight. (**C** and **D**) The expression of circCDC45 and miR-485-5p in the excised tissues was checked using qRT-PCR. (**E**) The expression of CSF-1 in the excised tissues was checked using western blot. *P < 0.05

We further investigated the role of circCDC45 in vivo. LN229 cells transfected with sh-circCDC45 or sh-NC were used for the tumorigenic test. The result showed that circCDC45 deficiency dramatically weakened tumor growth, including tumor volume and tumor weight (Fig. 7A and B). In the excised tumor tissues, we examined the expression of circCDC45, miR-485-5p and CSF-1. Compared to sh-NC, the expression of circCDC45 and CSF-1 was remarkably declined, while the expression of miR-485-5p was remarkably increased in the tissues from the sh-circCDC45 group (Fig. 7C, D and E). The data circCDC45 also played functions in vivo through the miR-485-5p/CSF-1 axis.

## Discussion

GBM is characterized by high invasion and reoccurrence**,** and frequent recurrence and invasion of GBM make it resistant to chemotherapy and radiation therapy [4]. GBM remains conferring poor outcomes with limited therapeutic progress. Biomarker-based projects have aroused much interest in precision medicine and targeted therapy [23]. Emerging evidence has identified circRNAs as novel biomarkers, and the investigation of specific circRNAs involved in tumorigenesis of GBM is imperative.

Previous studies showed that circFOXO3 enrichment accelerated GBM cell invasion by functioning as a “ceRNA” to mediate the miR-138-5p&miR-432-5p/NFAT5 network [24]. Circ_0029426 overexpression was linked to unfavorable prognosis of GBM, and circ_0029426 restoration induced GBM cell proliferation but blocked cell apoptosis [25]. High expression of CircSMARCA5 was linked to high overall survival, and circSMARCA5 regulated angiogenesis in GBM multiforme by binding to SRSF1 [26]. In our research, we investigated the function of circCDC45 in GBM and defined it as an oncogene. The evidence was that circCDC45 downregulation impaired GBM cell proliferation, invasion and migration in vitro and tumor development in vivo. CircCDC45 was monitored to be markedly upregulated in GBM tissues by circRNA microarray [11]. Besides, a recent study insisted that high expression of circCDC45 was closely linked to large tumor size, high grade and poor survival in glioma and contributed to glioma cell growth and metastasis [27]. All findings manifested that the inhibition of circCDC45 might be a novel strategy against GBM progression.

For mechanism analysis, we obtained potential target miRNAs of circCDC45, and miR-485-5p was indeed targeted by circCDC45 through multiple validations. A former study identified that miR-485-5p served as an anti-tumor role in glioma, and miR-485-5p overexpression glioma tumorigenesis by repressing the expression of its target gene TPD52L2 [15]. Nayak et al. discovered that restoration of miR-485-5p impaired GBM cell invasion and induced apoptosis [28]. Consistent with these findings, we discovered that the abundance of miR-485-5p was weak in GBM tissues and cells. MiR-485-5p inhibition counteracted the effects of circCDC45 downregulation to induce GBM cell proliferation, migration and invasion, while miR-485-5p restoration blocked these malignant behaviors. Similarly, miR-485-5p as a tumor suppressor was also recorded in other cancers, including hepatocellular carcinoma, cholangiocarcinoma and esophageal cancer [4, 29, 30], indicating that miR-485-5p was a well-recognized tumor suppressor in various cancers.

Furthermore, we noticed that miR-485-5p bond to CSF-1 3’UTR, and further assay verified that CSF-1 was indeed targeted by miR-485-5p. The tumorigenicity of CSF-1 was widely documented in various cancers, and high expression of CSF-1 predicted poor prognosis [31, 32]. Likewise in GBM, De et al. held the view that CSF-1 was frequently overexpression in human cancers, and overexpression of CSF-1 facilitated the formation of high-grade glioma [20]. Besides, CSF-1 overexpression promoted glioma cell viability and metastasis, while CSF-1 knockdown presented the opposite effects [33]. Consistent with these consequences, we viewed that the expression of CSF-1 was abnormally strengthened in GBM tissues and cells, and CSF-1 knockdown repressed GBM cell proliferation, invasion and migration. Additionally, CSF-1 expression was negatively linked to miR-485-5p expression but positively linked to circCDC45 expression in GBM tissues, and circCDC45 downregulation weakened CSF-1 expression, while miR-485-5p inhibition recovered CSF-1 expression, suggesting that circCDC45 regulated CSF-1 by targeting miR-485-5p.

## Conclusions

**Fig. 8:**
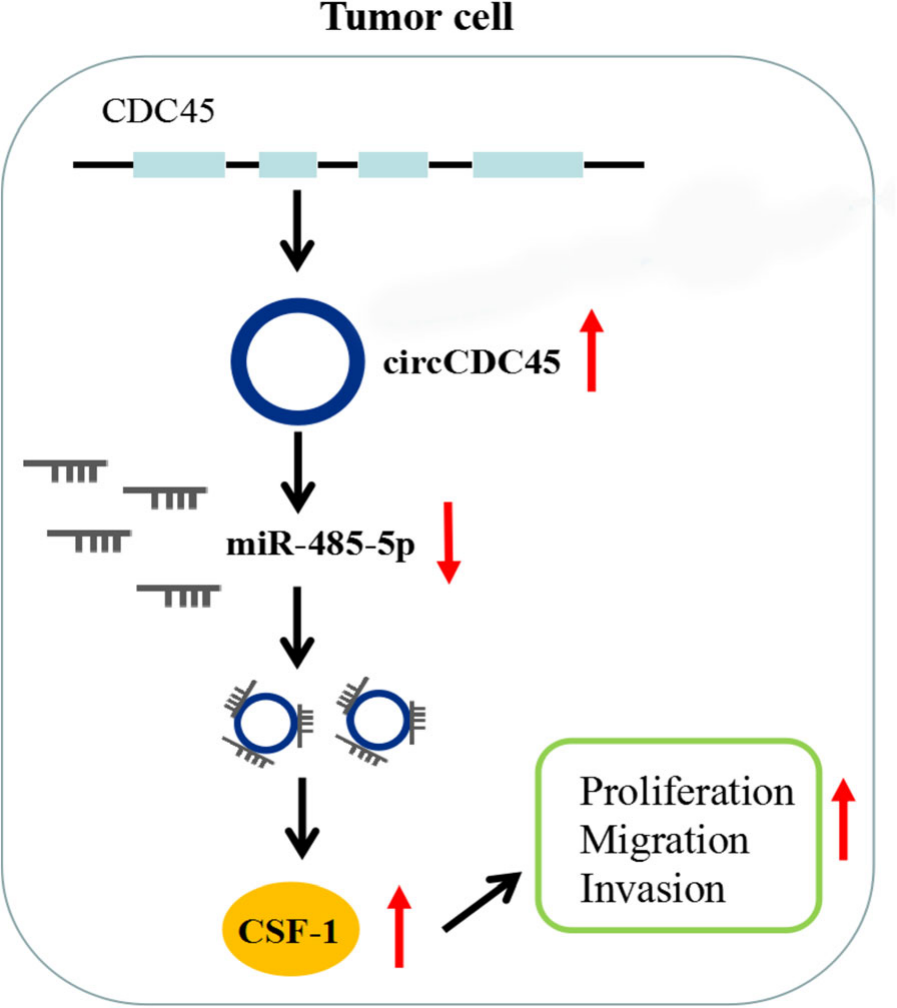
Schematic diagram revealed that the circCDC45/miR-485-5p/CSF-1 axis participated in the development of GBM

CircCDC45, derived from CDC45 mRNA, positively regulated the expression of CSF-1 by targeting miR-485-5p, thereby promoting GBM cell proliferation, migration and invasion and contributing to the progression of GBM (Fig. 8). Hence, circCDC45 was a promising biomarker for GBM diagnosis and treatment.

## Supplementary Information

**Additional file 1 Supplementary file**. The raw data of western blot for the expression levels of Ki67 and PCNA.

## Data Availability

All data generated or analysed during this study are included in this published article [and its supplementary information files].

## Abbreviations

GBM: Glioblastoma
circRNAs: Circular RNAs
CSF-1: Colony-stimulating factor 1
qRT-PCR: Quantitative real-time polymerase chain reaction
CCK-8: Cell counting kit − 8
RIP: RNA immunoprecipitation
circRNAs: Circular RNAs
ceRNAs: Competitive endogenous RNAs
miRNA: MicroRNA
MREs: MicroRNA response elements
3’UTR: 3′ untranslated regions
