# Supplementary figures and images for: CircCDC45 promotes the malignant progression of glioblastoma by modulating the miR-485-5p/CSF-1 axis
Source: BMC Cancer. 2021 Oct 9;21:1090. doi: 10.1186/s12885-021-08803-7 (PMC8501713; doi:10.1186/s12885-021-08803-7)

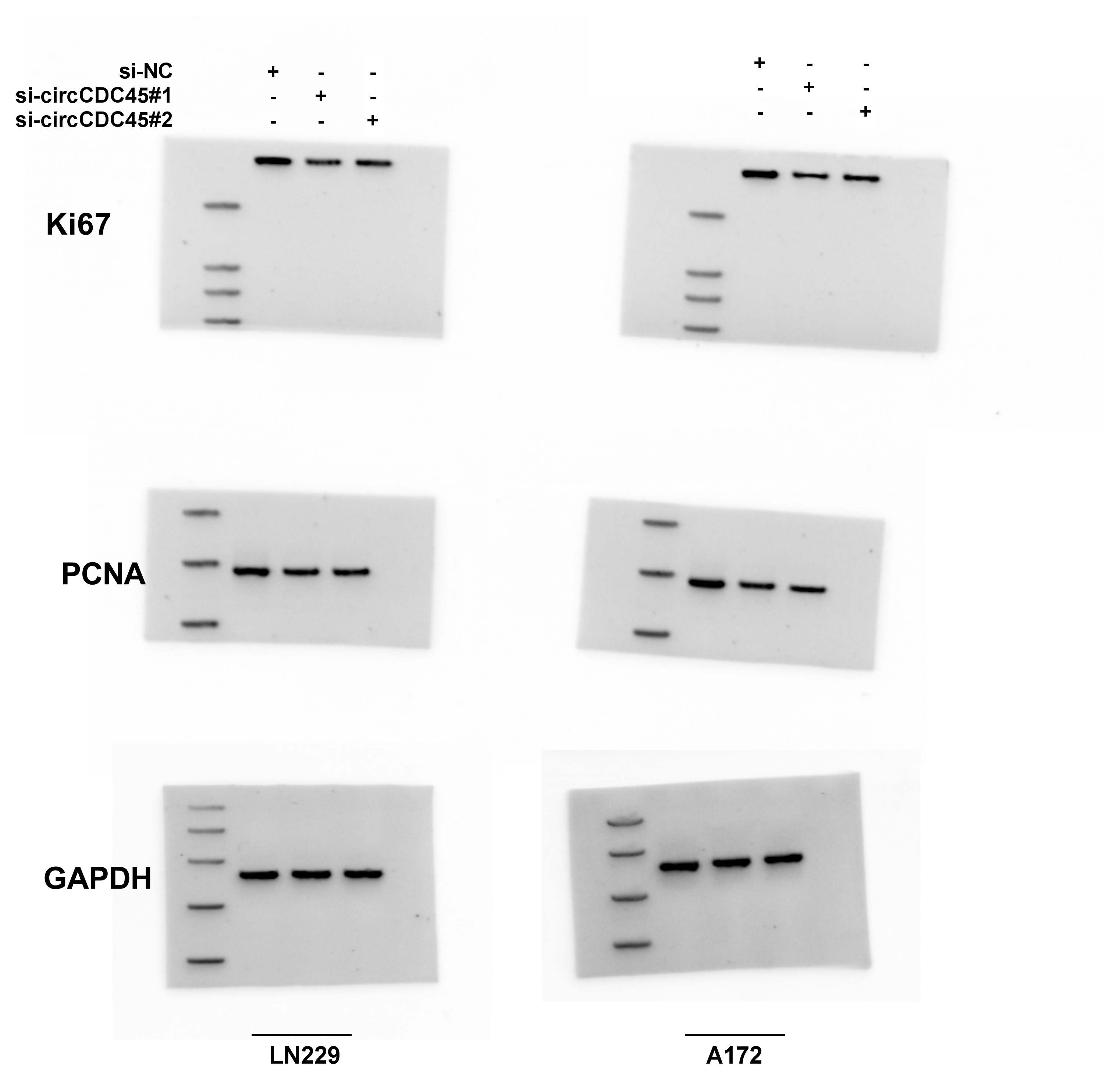

Supplement: Supplementary file 1 — Additional file 1 Supplementary file. The raw data of western blot for the expression levels of Ki67 and PCNA. [file 12885_2021_8803_MOESM1_ESM.zip › supplementary 1R4.tif]

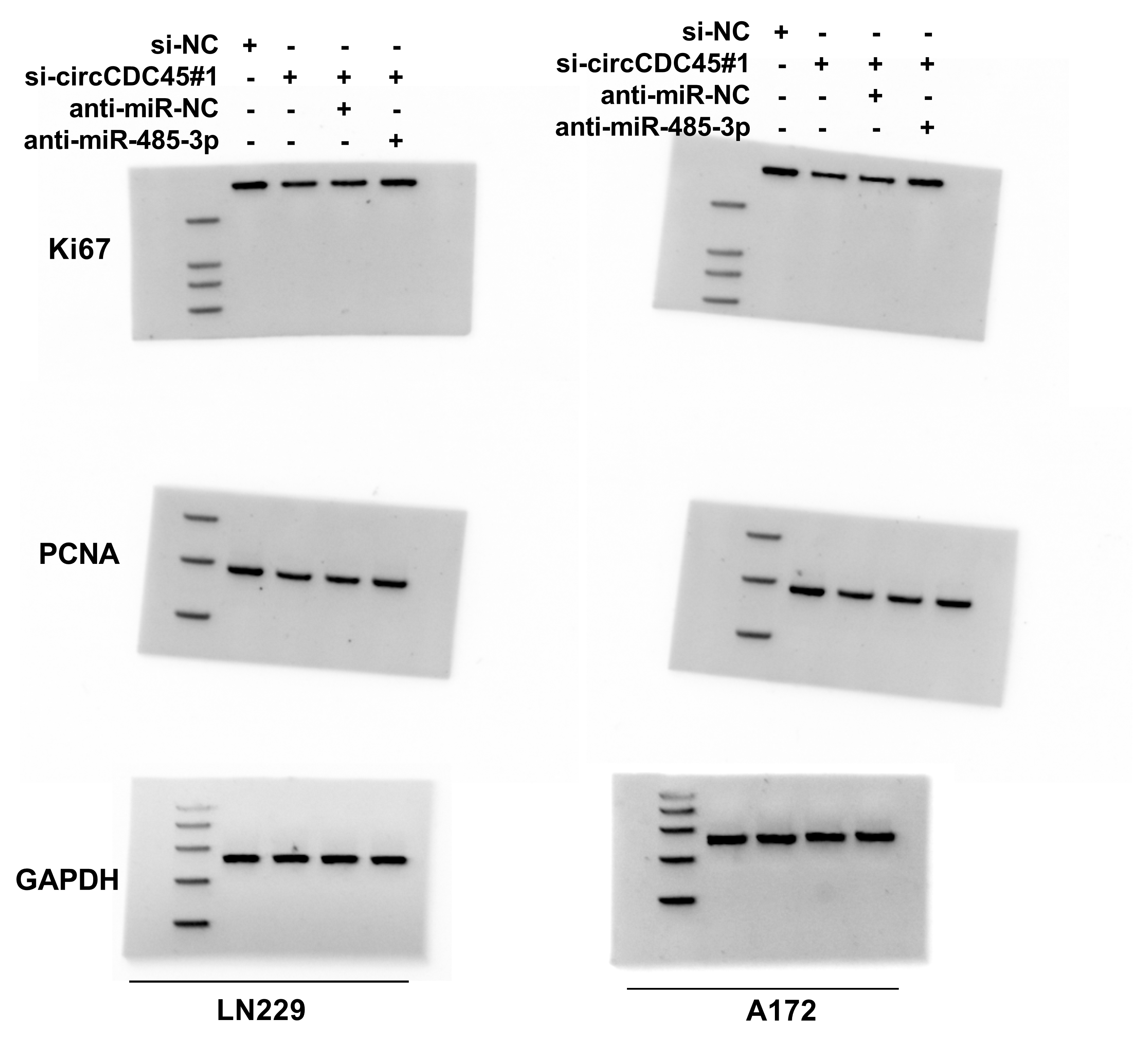

Supplement: Supplementary file 1 — Additional file 1 Supplementary file. The raw data of western blot for the expression levels of Ki67 and PCNA. [file 12885_2021_8803_MOESM1_ESM.zip › supplementary 2R4.tif]

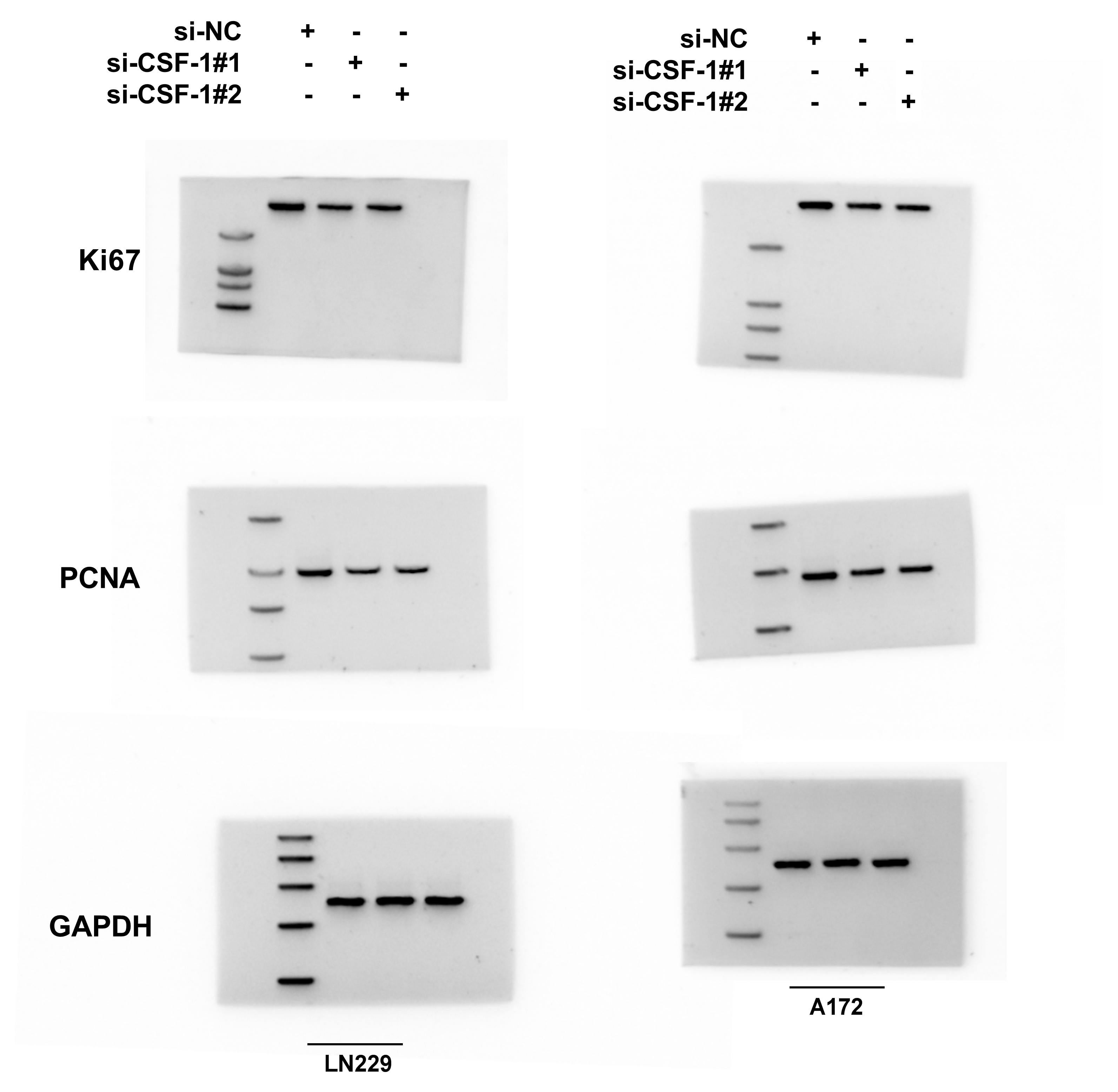

Supplement: Supplementary file 1 — Additional file 1 Supplementary file. The raw data of western blot for the expression levels of Ki67 and PCNA. [file 12885_2021_8803_MOESM1_ESM.zip › supplementary 3R4.tif]
